# Profiling and Discrimination of Euryale Ferox Seeds from Different Processing Methods Using Liquid Chromatography High-Resolution Mass Spectrometry Combined with Molecular Networking and Statistical Analysis

**DOI:** 10.3390/metabo15040225

**Published:** 2025-03-25

**Authors:** Xiaoyu Xie, Chuntao Zeng, Ruonan Zhang, Wenting Zhu, Huijie Li, Zhi Huang

**Affiliations:** 1 Department of Pharmacy, Jiangxi University of Chinese Medicine, Nanchang 330004, China; xiexiaoyu@jxutcm.edu.cn (X.X.); zengchuntao@jxutcm.edu.cn (C.Z.); zhangruonan1@jxutcm.edu.cn (R.Z.); zhuwenting1@jxutcm.edu.cn (W.Z.); lihuijie@jxutcm.edu.cn (H.L.); 2 School of Medical Laboratory, Hunan University of Medicine, Jinxi Road No.492, Huaihua 418000, China

**Keywords:** Euryale ferox seeds, processing, liquid chromatography high-resolution mass spectrometry, molecular networking, statistical analysis

## Abstract

Background: Euryale ferox seeds (EFSs) serve both medicinal and culinary purposes. They possess high nutritional value and are rich in polysaccharides, polyphenols, glycolipids, cyclic peptides, and other beneficial components. EFSs are known for their effects in tonifying the kidneys and strengthening essence, invigorating the spleen and alleviating diarrhea, as well as removing dampness and leucorrhea. Processing can alter the chemical composition of EFSs, with different methods yielding varying effects on their chemical makeup and, consequently, their efficacy. However, to date, no studies have systematically investigated the overall chemical composition of EFSs using different processing methods. Methods: In this study, we employed liquid chromatography high-resolution mass spectrometry (LC-HRMS) to identify the compounds in EFSs by searching databases and Global Natural Products Social Molecular Networking (GNPS), and we comprehensively explored the changes in the chemical composition of EFSs resulting from various processing methods via statistical analysis. Results: A total of 438 compounds were identified from EFSs, of which 283 were identified through database searches and 155 were identified via GNPS propagation. Statistical analysis revealed 32 and 38 differential compounds in dry-fried Euryale ferox seeds (DFEFSs) and bran-fried Euryale ferox seeds (BFEFSs), respectively. Additionally, we found a significant increase in the lipid content of the fried EFSs. Conclusions: This study provides valuable data to support the quality evaluation of processed EFSs and contributes to the research on the material basis of their medicinal efficacy.

## 1. Introduction

Euryale ferox seeds (EFSs) are a medicinal and edible resource with a long history in China [1,2]. EFSs are rich in bioactive compounds, including lignans, flavonoids, and tocopherols [3]. These compounds exert various effects, such as anti-aging, memory enhancement, and antidepressant properties, through mechanisms including antioxidation, anti-inflammation, and neuroprotection [4]. The chemical composition of EFSs is critical in determining their physiological functions. Processing is one of the characteristics of traditional Chinese medicine. Through processing, the physical and chemical properties of components in traditional Chinese medicine can be changed, thereby reducing or eliminating drug toxicity, transforming the medicinal properties, and enhancing the pharmacological effects [5]. The most used processing methods for EFSs are dry frying and bran frying [6]. However, there are currently no systematic reports on the chemical composition of EFSs after processing. Therefore, comprehensively detecting the chemical composition of EFSs and clarifying the changing rules of chemical components after processing can provide a basis for formulating processing technology parameters and quality standards.

Currently, methods for detecting compounds in EFSs include nuclear magnetic resonance (NMR), Fourier transform infrared spectroscopy (FT-IR), liquid chromatography with ultraviolet detection (LC-UV), and liquid chromatography high-resolution mass spectrometry (LC-HRMS) [7,8,9]. Among these techniques, NMR, FT-IR, and LC-UV exhibit low sensitivity, resulting in inadequate detection coverage. In contrast, LC-HRMS offers advantages such as high coverage, high sensitivity, and high throughput, making it widely utilized for the analysis of compounds in complex samples [10,11]. However, a review of the literature indicates that its application in EFSs is relatively limited, with more frequent use in detecting compounds from other tissues of the EF. For instance, Wu et al. identified nine compounds in purified anthocyanin extracts obtained from the waste leaves of EF using LC-HRMS [12]. Additionally, Wu et al. identified three phenolic compounds from the seed coat of EF [13]. Zhang et al. also identified seven phenolic compounds from the extract of the seed coat of EF [14]. However, the limited number of compounds in the MS database and the absence of automated tools for identifying compounds have resulted in a restricted number of compounds currently identified from EFSs, thereby hindering in-depth research on their biological activities. The Global Natural Products Social Molecular Networking (GNPS) not only identifies compounds in complex samples through its built-in database but also integrates compounds with similar MS/MS into the same network, facilitating classification and propagating the identification of compounds. It is widely used for exploring new compounds in complex samples [15,16,17]; however, its application in EFSs has not yet been reported.

In this study, we integrated database searching with GNPS to achieve a comprehensive identification of compounds in EFSs. First, we identified a subset of compounds in EFSs through database searching. Next, we classified the compounds in EFSs using GNPS, utilizing the database-identified results as seed compounds to facilitate propagating identification. Finally, based on the identification results from both database searching and GNPS, we employed various statistical methods to investigate the changes in EFSs after processing and identify potential processing markers. Our work provides a viable method for extensive compound identification and serves as a reference for exploring potential chemical markers in other herbal medicines.

## 2. Materials and Methods

### 2.1. Samples and Chemical Reagents

Raw Euryale ferox seeds (REFSs) were obtained from Jiangxi Minghu Agricultural Development Co., Ltd., Shangrao, China. The processing was conducted in accordance with the National Processing Standards for Chinese Materia Medica established in 1988 [18]. To produce dry-fried Euryale ferox seeds (DFEFSs), the raw EFSs were fried in a pan over low heat (80–100 °C) until their surfaces turned golden brown (for 15 min). REFSs possess are characteristically neutral to slightly cool, which make them potentially unsuitable for individuals with spleen and stomach deficiency-cold when consumed directly. However, during the bran-frying process, the warming properties of wheat bran partially counteract the cool nature of REFSs. As a result, bran-fried Euryale ferox seeds (BFEFSs) exhibit a milder medicinal character, effectively reducing gastrointestinal irritation. Therefore, the BFEFSs were prepared, and the production process is as follows: wheat bran was added to a preheated pan and heated over a medium flame (180–200 °C) until smoke appeared (for 0.5 min). The raw EFSs were then added, and the mixture was stir-fried continuously until the surface of EFSs turned golden brown (for 2 min). A quality control (QC) sample was created by combining equal amounts of EFSs from the different processing methods. A blank control sample was prepared using 90% ethanol in deionized water (*v*/*v*).

Acetonitrile (ACN) with HPLC grade was purchased from Merck (Darmstadt, Germany). Ultrapure water was generated using a Milli-Q system (Millipore, Billerica, MA, USA). Formic acid, ethanol, water, and ammonium acetate were purchased from Aladdin (Shanghai, China).

### 2.2. Preparation of Sample

EFS samples were ground into powders using a crusher (MM400, Miaokang, Jinhua, China), and then sifted through a 40-mesh filter sieve. Afterward, 0.6 g of EFS powder was mixed with 3 mL of 95% ethanol for ultrasonic extraction at 25 °C for 60 min. Subsequently, the solution was then filtered through a 0.22 μm membrane, and 1 mL of the filtrate was transferred to glass vials for LC-HRMS analysis.

### 2.3. LC-HRMS Analysis

The analysis was conducted using a Shimadzu 40A LC system (Shimadzu, Kyoto, Japan). The temperature of the ACQUITY BEH C18 column (2.1 mm × 100 mm, 1.7 μm, Waters, Milford, MA, USA) was maintained at 40 °C, while the sample manager temperature was set to 6 °C. A sample injection volume of 4 µL was utilized, and a flow rate of 0.3 mL/min was consistently maintained throughout the analysis. For positive ion mode, mobile phase A consisted of water with 0.1% formic acid, and mobile phase B was ACN. The elution gradient started at 10% B and was maintained for 1 min. Subsequently, it was linearly increased to 100% B over 23 min, which was then held for an additional 4 min. The system was then reverted to the initial ratio within 0.1 min and maintained for 2.9 min. For negative ion mode, mobile phase A was water containing 5 mM ammonium acetate, and mobile phase B was ACN. The elution gradient started at 5% B and was maintained for 1 min. Subsequently, mobile phase B was increased linearly from 5% to 95% over a period of 24 min and held at 95% for 3 min. Finally, the system was restored to the initial ratio within 0.1 min and maintained for 2.9 min.

Mass spectrometry detection was performed using a ZenoTOF 7600 MS system (SCIEX) in both positive and negative ion modes. The pressures for both gas 1 and gas 2 were set at 50 psi, and the source temperature was maintained at 500 °C. The declustering potential and spray voltage were set to 80 V and 5500 V, respectively. The mass spectrometry scan range for MS1 was 80–1250 Da. MS/MS acquisition was conducted using information-dependent acquisition (IDA) mode, with 18 candidate ions monitored per cycle. The *m*/*z* range for IDA was 50-1250 Da, and the energy for collision-induced dissociation was set at 30 ± 15 eV.

### 2.4. Data Preprocessing and Statistical Analysis

Molecular network was constructed using the GNPS platform and visualized with Cytoscape 3.9.1 software. The raw data generated by LC-HRMS were converted to the “mgf” format using MSConvert and subsequently uploaded to the GNPS online platform. The GNPS parameters were set as follows: Both the precursor ion mass tolerance and the fragment ion tolerance were set to 0.02 Da. Clusters were composed of at least 2 ions. The matching condition was defined as a score greater than 0.6, with a minimum of 3 matching peaks.

MS-DIAL software (version 5.2.240424) was utilized for data collection, peak detection, compound identification, peak filtering, and peak alignment. The identification results of MS-DIAL were subjected to manual confirmation. The detailed parameter settings were performed as previously described [19].

Principal component analysis (PCA) and partial least squares–discriminant analysis (PLS-DA) were performed using SIMCA-P 13 software (Umetrics, Umea, Sweden). The volcano plots were generated using GraphPad Prism 8.0 software (GraphPad, La Jolla, CA, USA). The heat map was created with the online platform Omicstudio (https://www.omicstudio.cn (accessed on 17 December 2024)).

## 3. Results and Discussion

### 3.1. Identification of Chemical Constituents in EFSs

Combined with the identification results of searching databases and propagating identification, a total of 438 compounds were identified, including 88 fatty acids and their derivatives, 78 glycerophospholipids, 57 carbohydrates and their conjugates, 37 flavonoids and their derivatives, 37 fatty amines, 24 lignans, 25 terpenoids and their derivatives, 22 glycerolipids, 5 cinnamic acids and their derivatives, 5 sphingolipids, and 60 other compounds (Table S1). Within the 438 compounds, 283 compounds were identified by searching databases, and 155 compounds were identified by propagated identification.

#### 3.1.1. Identification by Searching Databases

To provide a more detailed explanation of the identification process for searching databases, we randomly selected specific compounds from classes that contain a substantial number for illustration.

##### Identification of Fatty Acids and Derivatives

**Figure 1:**
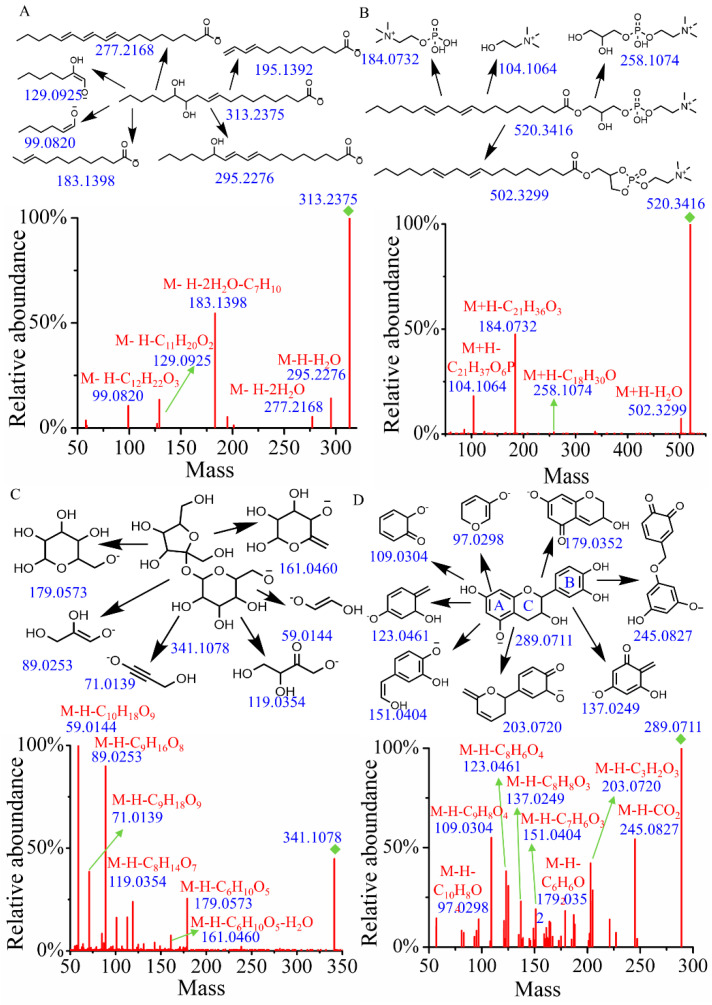
The fragment structure and MS/MS of compounds. (**A**) 12(13)-DiHOME. (**B**) (2-Hydroxy-3-octadeca-9,12-dienoyloxypropyl) 2-(trimethylazaniumyl)ethyl phosphate. (**C**) Hex-2-ulofuranosyl hexopyranoside. (**D**) Epicatechin.

Fatty acids, particularly hydroxylated fatty acids, are prone to generate neutral loss of water and cleavage of alkyl chains [20]. As shown in Figure 1A, when searching the MSMS_Public_EXP_VS17 database using both MS1 and MS/MS, the identification result was 12(13)-DiHOME, with a similarity score of 0.87. To verify this identification, a structural annotation of MS/MS spectrum was performed. Notably, the fragments at *m*/*z* 295.2276 and 277.2168 were attributed to the loss of H_2_O and 2H_2_O from the quasi-molecular ion (*m*/*z* = 313.2375), respectively. The fragment at *m*/*z* 183.1398 (M-H-2H_2_O-C_7_H_10_) resulted from the sequential loss of 2H_2_O and 2-heptene alkyl. Additionally, the fragment ions at *m*/*z* 129.0925 and 99.0820 were assigned as [M-H-C_11_H_20_O_2_]^−^ and [M-H-C_12_H_22_O_3_]^−^, respectively. Therefore, the main fragmentation structures have been reasonably assigned, and the peak at t_R_ = 11.53 and *m*/*z* = 313.2375 was tentatively identified as 12(13)-DiHOME.

##### Identification of Glycerophospholipids

Glycerophospholipids typically undergo specific cleavage of the phosphate head group, the glycerol backbone, and the fatty acid ester bonds [21]. In positive mode, a precursor ion [M+H]^+^ at *m*/*z* 520.3416 and t_R_ = 15.61 min was identified. A search of the MSMS_Public_EXP_VS17 database revealed that it was (2-hydroxy-3-octadeca-9,12-dienoyloxypropyl) 2-(trimethylazaniumyl) ethyl phosphate with a score of 0.92 (Figure 1B). The neutral loss of H_2_O from the quasi-molecular ion generated a fragment ion at *m*/*z* 502.3299. Following the cleavage of the fatty acid ester bonds, a fragment ion at *m*/*z* 258.1074 was produced. Additionally, the characteristic ion at *m*/*z* 184.0732, resulting from the cleavage of the phosphorylcholine head group, and the ion at *m*/*z* 104.1064 from the glycerol backbone were also observed in the MS/MS. Accordingly, compound 64 was tentatively identified as (2-hydroxy-3-octadeca-9,12-dienoyloxypropyl) 2-(trimethylazaniumyl) ethyl phosphate.

##### Identification of Carbohydrates and Conjugates

In the electrospray ionization process under negative ion mode, glycosidic bond cleavage and the neutral loss of H_2_O typically occur in carbohydrates and their conjugates [22]. Compound 9 (t_R_ = 0.91 min), which gave the [M-H]^−^ ion at *m*/*z* 341.1078, was identified as hex-2-ulofuranosyl hexopyranoside by searching databases with a score of 0.94. As illustrated in Figure 1C, the fragment ion with *m*/*z* of 179.0573 (M-H-C_6_H_10_O_5_) results from the cleavage of the glycosidic bond, accompanied by the neutral loss of 2,5-anhydro-D-mannitol. Meanwhile, the fragment ion with an *m*/*z* of 161.0460 (M-H-C_6_H_10_O_5_-H_2_O) is produced from a further neutral loss of H_2_O following the neutral loss of 1,5-anhydrohexitol. The parent ion with *m*/*z* of 179.0573 undergoes additional fragmentation, resulting in fragments with *m*/*z* values of 119.0354, 89.0253, 71.0139, and 59.0144, which are assigned as [M-H-C_8_H_14_O_7_]^−^, [M-H-C_9_H_16_O_8_]^−^, [M-H-C_9_H_18_O_9_]^−^, and [M-H-C_10_H_18_O_9_]^−^, respectively. Combined with database alignment, compound 9 was tentatively identified as hex-2-ulofuranosyl hexopyranoside.

##### Identification of Flavonoids and Derivatives

The most common characteristic of flavones and their derivatives is the occurrence of retro-Diels–Alder (RDA) fragmentation [23]. Compound 289 (t_R_ = 5.10 min, *m*/*z* = 289.0711) was identified as epicatechin by searching the database. As illustrated in Figure 1D, the peaks at *m*/*z* 137.0249 (M-H-C_8_H_8_O_3_) and 151.0404 (M-H-C_7_H_6_O_3_) correspond to the characteristic fragments resulting from RDA fragmentation. The fragment ion at *m*/*z* 245.0827 is produced by the neutral loss of CO_2_ from the quasi-molecular ion peak. The fragment at *m*/*z* 203.0720 (M-H-C_3_H_2_O_3_) arises from cleavage in the A ring, while the fragment at *m*/*z* 123.0461 (M-H-C_8_H_6_O_4_) originates from cleavage of the C ring. The fragment at *m*/*z* 179.0352 (M-H-C_6_H_6_O_2_) is generated by the bond breakage between the B and C rings. Additionally, the fragment at *m*/*z* 109.0304 corresponds to [M-H-C_9_H_8_O_4_]^−^ and the fragment at *m*/*z* 97.0298 corresponds to [M-H-C_10_H_8_O_4_]^−^.

#### 3.1.2. Identification Through Propagation

**Figure 2:**
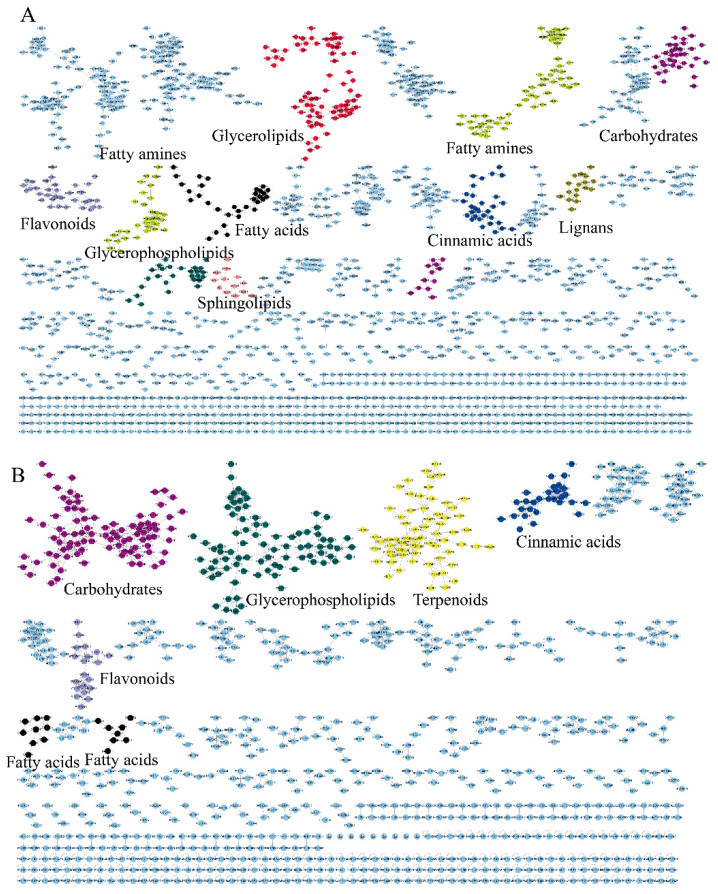
The molecular networks of EFSs. (**A**) Positive ion mode. (**B**) Negative ion mode.

To achieve propagating identification, a molecular network based on MS/MS similarity was constructed (Figure 2). The positive model comprised 6256 precursor ions, organized into 323 clusters and 4810 single nodes (Figure 2A). The negative model included 3448 precursor ions, comprising 158 clusters and 2625 single nodes (Figure 2B). The molecular network reveals that fatty acids, glycerophospholipids, carbohydrates and carbohydrate conjugates, flavonoids and their derivatives, fatty amines, lignans, terpenoids and their derivatives, glycerolipids, cinnamic acids and their derivatives, and sphingolipids each form distinct clusters.

**Figure 3:**
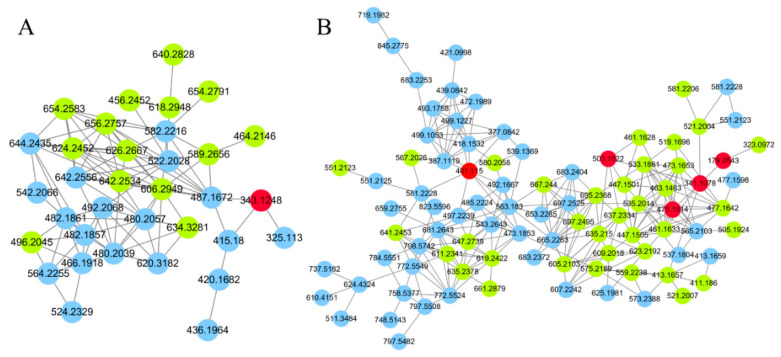
The molecular networks of carbohydrates and conjugates. (**A**) Positive ion mode. (**B**) Negative ion mode. The red nodes indicate identification through database search, the green nodes indicate identification through propagation by GNPS, the blue nodes indicate unknow compounds.

Since GNPS can only provide automated annotation for a limited subset of compounds through its built-in databases, compounds that cannot be automatically identified require propagating identification under the guidance of seed compounds (structurally known compounds). In this study, the seed compounds are the identification results obtained from searching databases using MS-DIAL and GNPS. For example, the clusters of carbohydrates and their conjugates are illustrated in Figure 3. The red nodes, identified as carbohydrates through database searches, indicate that other nodes within the same cluster also belong to the carbohydrate. Based on this, the structures of the non-red node compounds were further identified by matching their MS1 data with the COlleCtion of Open Natural prodUcTs database with self-written Python code (Version 3.9). As a result, under the positive ion mode and negative ion mode, 14 and 36 compounds (green nodes) were successfully annotated, respectively.

### 3.2. Discrimination of EFSs from Different Processing Methods by Multivariate Statistical Analysis

**Figure 4:**
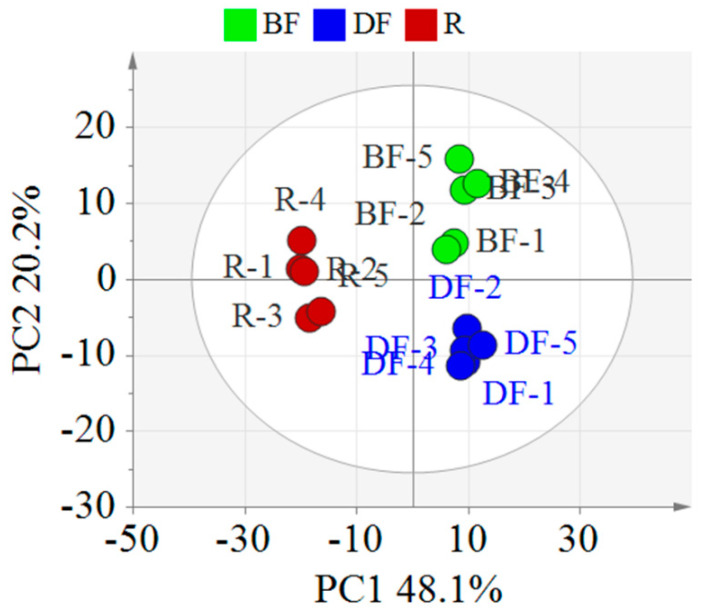
PCA score plot of different EFSs. R represents raw Euryale ferox seeds, DF represents dry-fried Euryale ferox seeds, and BF represents bran-fried Euryale ferox seeds.

To clarify the differences in the chemical components of EFSs under various processing methods, this study employed PCA for data analysis (Figure 4). The PCA score plot revealed that the samples of the REFSs, DFEFSs, and BFEFSs clustered distinctly, indicating disparities in their chemical compositions. Notably, REFSs are located in the second and third quadrants, while DFEFSs and BFEFSs are situated in the first and fourth quadrants. This suggests that the compounds of the REFS group differ significantly from those of the DFEFS and BFEFS groups. Therefore, the conditions of processing have a significant influence on the compounds of EFSs.

#### 3.2.1. Statistical Analysis Between REFSs and DFEFSs

**Figure 5:**
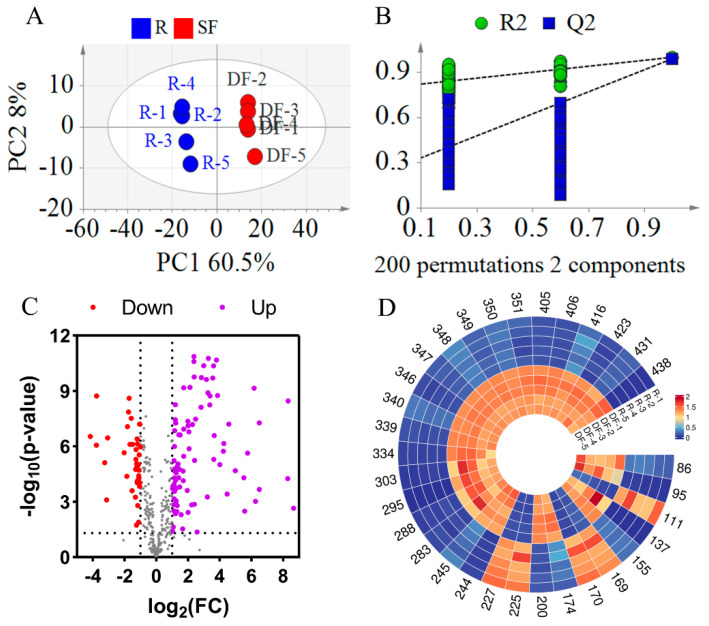
Statistical analysis results of DFEFS and REFS groups. (**A**) PLS-DA score plots. (**B**) PLS-DA model permutation test. (**C**) Volcano plot. (**D**) Heatmap of potential chemical markers. R represents raw Euryale ferox seeds and DF represents dry-fried Euryale ferox seeds.

To more precisely identify the compounds responsible for the differences between REFSs and DFEFSs, PLS-DA was employed. As shown in Figure 5A, the R^2^X, R^2^Y, and Q^2^ values were 0.685, 0.998, and 0.989, respectively, indicating the excellent reproducibility and predictive capability of the model. Additionally, a permutation test with 200 iterations was carried out (Figure 5B). The Q^2^ [0.0, 0.257] and R^2^ [0.0, 0.801] were lower than the original values. This suggests that there are no issues with randomness or overfitting in the model, further confirming its reliability. In addition, a clear separation trend was observed between the REFS and DFEFS groups, further indicating that significant changes occurred in the compounds following the dry-frying processing of EFSs.

A volcano plot was constructed based on *t*-test and fold change (FC) (Figure 5C). This plot reveals that the content of most compounds increased after dry-frying. Using PLS-DA, compounds with a Variable Importance in Projection (VIP) greater than 1.2 were selected. Additionally, compounds with an FC greater than 4 or less than 0.25, along with p less than 0.05, were screened. By integrating the differential compounds from the *t*-test, FC, and multivariate analyses, 32 overlapping compounds were selected (Table S2, Figure 5D). Among these 32 differential compounds were 4 glycerophospholipids (Compounds 86, 95, 111, and 137), 7 fatty acids and their derivatives (Compounds 155, 169, 170, 174, 200, 225, and 227), 2 fatty amines (Compounds 244 and 245), 4 flavonoids and their derivatives (Compounds 283, 288, 295, and 303), 9 glycerolipids (Compounds 334, 339, 340, 346, 347, 348, 349, 350, and 351), and 6 other compounds (Compounds 405, 406, 416, 423, 431, and 438). Furthermore, of the 32 compounds, 22 were classified as lipids. Among these 22 lipid compounds, 17 exhibited increased content after DF. These differential compounds hold significant potential for distinguishing between REFSs and DFEFSs.

#### 3.2.2. Statistical Analysis Between REFSs and BFEFSs

**Figure 6:**
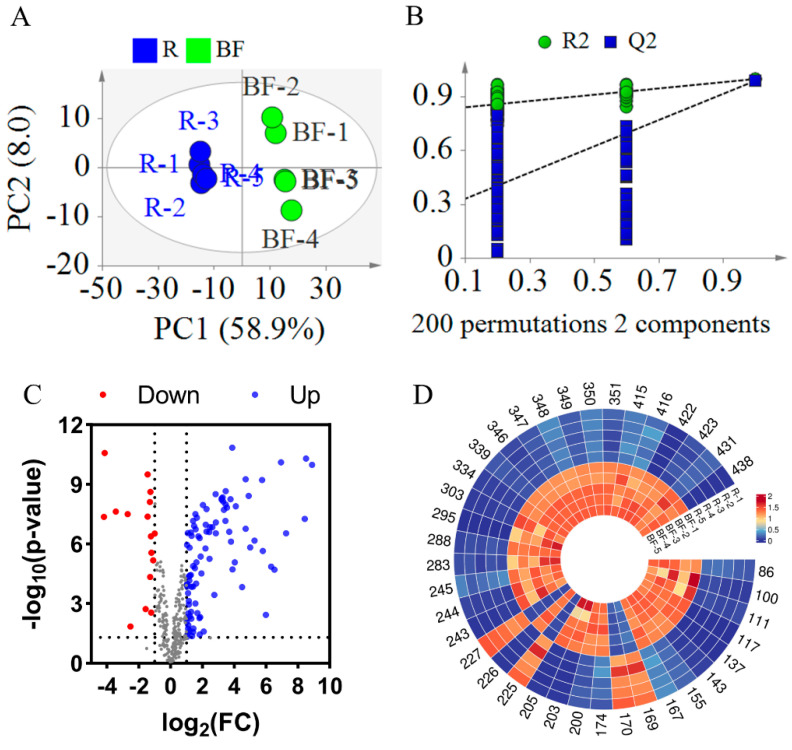
Statistical analysis result of BFEFS and REFS groups. (**A**) PLS-DA score plots. (**B**) PLS-DA model permutation test. (**C**) Volcano plot. (**D**) Heatmap of potential chemical markers. R represents raw Euryale ferox seeds and BF represents bran-fried Euryale ferox seeds.

Similarly, to investigate the changes in compounds in EFSs after bran frying, a PLS-DA model was established. As illustrated in Figure 6A, the R^2^X, R^2^Y, and Q^2^ values were 0.669, 0.999, and 0.989, respectively, indicating a good fit and predictability of the model (Figure 6A). To validate the PLS-DA model, a 200-iteration permutation test was conducted. As shown in Figure 6B, the Q^2^ and R^2^ were [0.0, 0.256] and [0.0, 0.823], respectively, which are lower than the original values. This indicates that the PLS-DA mode is not overfitting. In Figure 6A, the samples of REFSs and BFEFSs were in different areas, revealing that BF significantly altered the compounds in EFSs.

The *t*-test and FC calculation were employed to screen out significantly differential compounds with an FC greater than 4 or less than 0.25. Based on the screening results, a volcano plot was generated. As illustrated in Figure 6C, the majority of compounds exhibited increased levels following the bran-fried processing of EFSs. By integrating the *t*-test, FC, and multivariate analysis (PLS-DA), a total of 38 significantly differential compounds were ultimately screened (see Table S3 and Figure 6D). Among these 38 differential compounds, there were 5 glycerophospholipids (Compounds 86, 100, 111, 117, and 137), 12 fatty acids and their derivatives (Compounds 143, 155, 167, 169, 170, 174, 200, 203, 205, 225, 226, and 227), 3 fatty amines (Compounds 243, 244, and 245), 4 flavonoids and their derivatives (Compounds 283, 288, 295, and 303), 8 glycerolipids (Compounds 334, 339, 346, 347, 348, 349, 350, and 351), and 6 other compounds (Compounds 415, 416, 422, 423, 431, and 438). Notably, of the 38 compounds, 28 were classified as lipids. Among these 28 lipid compounds, 22 demonstrated increased content after DF. As shown in Tables S2 and S3, 28 compounds were identified as common differential compounds between DFEFSs and BFEFSs, with 20 of these being lipids. Intriguingly, among these 28 common differential compounds, all except PG 14:0_16:0 exhibited consistent trends in content changes after frying. When combined with the results from Figure 4, this suggests that the differences in compounds between DFEFSs and BFEFSs primarily stem from specific variations in content rather than their composition.

Overall, we found that the lipid content in FEFSs increased significantly. This may be attributed to several factors: Firstly, high-temperature frying can disrupt plant cell walls, facilitating the release of lipids from the cells [24]. Secondly, in raw medicinal materials, certain lipids form complexes with proteins and polysaccharides through enzymatic catalysis. When high temperatures destroy enzyme activity, the stability of these complexes diminishes, leading to the conversion of bound lipids into free lipids, which results in a marked increase in the detected values [25]. Thirdly, lipases, esterases, and other enzymes naturally present in traditional Chinese medicines may play a role in the decomposition and metabolism of lipids. High-temperature frying denatures and deactivates these enzyme proteins, thereby inhibiting the hydrolysis of lipids [26].

## 4. Conclusions

In the present study, we conducted a comprehensive analysis of the compound composition of EFSs and the changes in compounds resulting from various processing methods. Utilizing databases and GNPS, we identified a total of 438 compounds, of which 283 were identified through database searches, while 155 compounds were identified via propagated identification. Statistical analysis revealed that, compared to REFSs, the contents of 32 and 38 compounds changed significantly in DFEFSs and BFEFSs, respectively. Notably, among these compounds with significant changes, the majority of lipids exhibited increased levels. The findings of this study can be used to differentiate EFSs based on processing methods, establishing a solid foundation for further quality control and providing a theoretical basis for evaluating safety and nutritional aspects. Further research is necessary to elucidate the mechanisms underlying the transformation of chemical constituents across various processing methods, as well as to investigate the pharmacological effects of differently processed EFSs.

## Data Availability

The original contributions presented in this study are included in the article/supplementary material. Further inquiries can be directed to the corresponding author.

## Supplementary Materials

The following supporting information can be downloaded at https://www.mdpi.com/article/10.3390/metabo15040225/s1, Table S1: Detailed information of 438 compounds identified by searching databases and GNPS; Table S2: 32 compounds exhibited significant changes between DFEFSs and REFSs; Table S3: 38 compounds exhibited significant changes between BFEFSs and REFSs.
